# Cincinnati Board of Health

**Published:** 1883-08

**Authors:** 


### CINCINNATI BOARD OF HEALTH.

The Cincinnati *Lancet and Clinic*, speaking of a brand new board of health just elected for that city by the city fathers, says of the only doctor (so-called) on the board. He is one of the most notorious quacks that infests the city, a frequent patron of saloons, advertises to cure the opium habit, to restore lost virginity, etc :

Note the pedigree and occupations of the already famous Cincinnati Board of Health. Five saloon keepers and one poor, blear eyed quack doctor that wears a chronic blush on his nasal organ. Just why so much medicated wisdom was injected into that board is beyond our ken.

Our boy says its all right. The cholera is coming and that will make business good for the doctors. The five saloon keepers can out-vote the other fellow every time, while they will have the advantage of his advice and operative skill in case their virtue is assailed.

[Thus it ever is, the poor people must ever suffer that a ring of pot-house politicians may flourish and prosper. When will the people learn to protect themselves at the ballot box ?—Ed.]

